# Prevalence of malaria and lymphatic filariasis in *bateyes* of the Dominican Republic

**DOI:** 10.1186/s40249-019-0547-3

**Published:** 2019-05-27

**Authors:** Hunter M. Keys, Gregory S. Noland, Madsen Beau De Rochars, Stephen Blount, Manuel Gonzales

**Affiliations:** 1 Department of Anthropology, University of Amsterdam, Building B-REC B 8.01, Nieuwe Achtergracht 166, 1018 WV Amsterdam, The Netherlands; 2 The Carter Center, 453 Freedom Parkway, Atlanta, GA 30307 USA; 3 Health Services Research, Management and Policy Department, University of Florida, 1225 Center Drive, HPNP 3101, Gainesville, FL 32611 USA; 4 Center for Prevention and Control of Vector-borne and Zoonotic Diseases, Av. Juan Pablo Duarte No. 269, 10301 Santo Domingo, Dominican Republic

**Keywords:** Dominican Republic, Haiti, Malaria, Lymphatic filariasis, Elimination, Migrant

## Abstract

**Background:**

The island of Hispaniola, shared by Haiti and the Dominican Republic (DR), is the only remaining malaria-endemic island in the Caribbean and accounts for 95% of the lymphatic filariasis (LF) burden in the Americas. Both countries aim to eliminate the diseases by 2020. Migration from Haiti, where both diseases are more prevalent, may promote transmission in the DR. Historically, Haitian migrant labourers live in rural Dominican agricultural ‘company towns’ called *bateyes,* many of which received mass drug administration (MDA) for LF elimination. This study sought to determine the prevalence of malaria and LF in *bateyes* of the DR and to describe related risk factors for disease.

**Methods:**

From March to April 2016, a cross-sectional, cluster survey was conducted across Dominican *bateyes* stratified into three regions: southwest, north and east. A household questionnaire (*n* = 776), captured demographics, ethnic origin, mobility patterns, malaria intervention coverage, and knowledge, and recent fever and treatment-seeking. Two individuals per household (*n* = 1418) were tested for malaria parasites by microscopy and rapid diagnostic test (RDT) and LF antigen by filariasis test strip (FTS). Population-level estimates and confidence intervals (CI) were computed adjusting for the survey design. Two-sided *t*-tests compared differences in knowledge scores.

**Results:**

No (0%) blood sample was *Plasmodium*-positive by microscopy or RDT. Six individuals were FTS-positive (0.5%; 95% *CI*: 0.2–1.5), but none (0%) of these were microfilariae-positive. Most *batey* residents were born in the DR (57.8%), documented (85.0%), and permanent residents (85.1%). Very few respondents (9.4%) reported travel to Haiti in the past year. Overall, half (53.8%) of respondents owned a bed net, and 82.3% of net owners reported using it the previous night. Indoor residual spraying (IRS) differed by region (range: 4.7%–61.2%). Most of those with recent fever sought care (56.0%), yet only 30.5% of those seeking care were tested for malaria. Compared to Dominican-born populations, Haitian-born respondents more frequently reported recent fever, did not seek care for the fever, had not heard of malaria, and could not name symptoms or prevention methods.

**Conclusions:**

Malaria and LF transmission appear absent or extremely low in Dominican *bateyes*, which are a mixture of Haitian and Dominican residents. Travel to Haiti is rare, meaning risk of malaria and LF importation is low. Addressing identified gaps in intervention coverage, malaria knowledge, treatment seeking and service delivery will improve the quality of surveillance for these diseases, particularly among marginalized populations and promote island-wide elimination.

**Electronic supplementary material:**

The online version of this article (10.1186/s40249-019-0547-3) contains supplementary material, which is available to authorized users.

## Multilingual abstract

Please see Additional file 1 for translations of the abstract into five official working languages of the United Nations.

## Background

Malaria and lymphatic filariasis (LF) are mosquito-borne, parasitic diseases responsible for significant morbidity and mortality globally and disproportionately affect the poor [1–3]. The island of Hispaniola, shared by Haiti (population, 10.6 million) and the Dominican Republic (DR) (population, 10.7 million), is the only remaining malaria-endemic island in the Caribbean and accounts for around 95% of LF cases in the Western hemisphere [4–6]. In Hispaniola, malaria is caused by *Plasmodium falciparum* transmitted via *Anopheles albimanus* mosquitoes. The parasite remains chloroquine sensitive [7], with chloroquine plus primaquine the first-line treatment for uncomplicated malaria in both countries. LF in Hispaniola is caused by *Wuchereria bancrofti*, thin parasitic worms that inhabit the afferent lymphatic vessels and that are transmitted by *Culex quinquefasciatus* mosquitoes [4]. Infection leads to lymphatic dysfunction that can result in severe swelling in lower extremities (lymphedema) or male scrotum (hydrocele). In 2009, Haiti and the DR announced a binational plan to eliminate malaria from the island by 2020 [8]—the same date targeted for global LF elimination.

The strong association between poverty and these diseases is apparent when considering that Haiti, one of the poorest countries in the world [9], bears the largest share of both diseases on the island. Malaria is one of the ten principal causes of death in Haiti [10] and over 17,000 cases (approximately 97% of Hispaniola’s total) were reported annually from 2013−2016 [11]. In contrast, the DR has reported less than 1000 malaria cases and less than ten deaths annually since 2012 [11]. Transmission occurs year-round in both countries with minor seasonal peaks in January and in July. LF also disproportionately affects Haiti: a nation-wide survey in 2001 revealed that 88% of the country’s districts (*communes*) were considered endemic [12]. The Ministry of Public Health and Population targeted the entire country for annual mass drug administration (MDA) with diethylcarbamazine citrate (DEC; donated by Eisai since 2013) and albendazole (donated by GlaxoSmithKline) [13]. As of 2018, 84% of communes have qualified to stop MDA [14]. In the DR, baseline LF mapping using a school-based lot quality assurance sampling (LQAS) approach [15] identified 19 endemic municipalities clustered into three geographic foci: the Southwest, the East and in La Ciénaga, a small impoverished neighborhood of the capital, Santo Domingo [16]. The national LF elimination program (*Programa de Eliminación de la Filariasis Linfática*, PELF) sequentially scaled-up MDA in each foci beginning in 2002, with implementation units (IU) differing across each region due to administrative and endemicity boundaries: the Southwest focus IUs (municipalities) received five rounds of annual DEC-albendazole MDA between 2002−2007, La Ciénaga IUs (*sub-barrios*) received three rounds between 2004−2006, and the East IUs (*bateyes*) received three rounds between 2014−2017. As of 2018, all three areas stopped MDA for LF and are in post-treatment surveillance.

Historically, malaria and LF in the DR have occurred along the border with Haiti and in agricultural regions populated by migrant labourers, suggesting importation from Haiti [17, 18]. Since the early 20^th^ century, Haitian labourers were recruited *en masse* to work on sugar plantations, living in adjacent ‘company towns’ called *bateyes* [19]. Over time, *batey* populations became a complex admixture of Haitian migrants, Dominican-born children of the migrants, and Dominicans searching for employment in the sugar industry. A porous border and discriminatory policies in the DR leave migrants and their descendants vulnerable to human rights abuses, particularly in regards to obtaining authorized status [20]. For example, the 2013 Constitutional *Sentencia* effectively stripped entire generations of mostly Haitian-descended Dominicans of their citizenship [21]. With the decline of the sugar industry, *bateyes* are currently some of the poorest communities in the DR [22].

The distribution of malaria and LF varies considerably not just between the two countries but within them. In low-transmission settings such as the DR, remaining parasite reservoirs tend to be clustered among marginalized social groups such as migrants and the rural poor; reaching these groups and maintaining ongoing surveillance is crucial to elimination [23, 24]. However, the situation in the DR is complicated by a migration pattern that involves a largely undocumented population moving from sending communities with higher likelihood of disease prevalence to receiving communities where healthcare and surveillance may be inadequate. Given reports of high asymptomatic malaria infection in parts of Haiti [25], it is especially important to monitor disease prevalence in the DR in areas with high concentration of migrants. These epidemiological features transpire against a backdrop of charged political debates over how migrants and their descendants should (or should not) be recognized in Dominican society [20]. In a setting of already limited material resources, these socio-political realities challenge efforts to expand services to the population.

The purpose of this study was to assess malaria and LF prevalence in Dominican *bateyes*, and to describe *batey* residents’ demographics, mobility patterns, disease-specific knowledge and prevention practices, healthcare utilization, and barriers to care. This information is critical to the design of interventions to eliminate these two diseases in the DR and across Hispaniola.

## Methods

### Study design

The study design was a cross-sectional multi-stage cluster survey conducted from 9 March to 24 April, 2016. This period roughly corresponds to the end of the *zafra*, or annual sugar cane harvest season, when the migrant labour population is thought to be largest.

A target sample size of 1446 individual blood samples was sufficient to detect a 5% prevalence of malaria and LF with absolute precision of ± 2.5% at the 95% two-sided significance level, a design effect of 1.5, and a 10% non-response rate. To obtain this target sample size, a sampling frame was generated using a 2012 nationwide census of *bateyes*, which tend to be concentrated in four regions: Southwest, East, North, and greater capital area [26]. After excluding the capital region, which was thought to be less reliant on migrant labour due to its waning importance in agricultural production, the survey designated the Southwest, East, and North regions as individual strata to obtain representative results within each region.

Across the three regions, a total of 51 clusters (*bateyes*), or 17 *bateyes* within each region, were selected from a random start using probability of selection proportional to population size (PPS). Several larger *bateyes* were selected more than once, in which case the *batey* comprised two (or in one case, three) clusters. Within each cluster, 15 households were systematically selected from a random start using a sketch map of households made by field staff before surveying activities.

### Data collection

The survey consisted of two main components: a household-level questionnaire and blood sample testing. At each household, the adult (> 18 years old) head-of-household or his/her spouse was asked to complete a survey questionnaire. The questionnaire included ethnicity (Haitian-born, Dominican-born with Haitian descent, or Dominican-born without Haitian descent), basic demographics, mobility patterns, fever and illness experiences, treatment-seeking behaviour, knowledge and practices regarding malaria, LF, and diabetes, and perceptions of interpersonal discrimination. The findings from the diabetes and discrimination modules will be reported elsewhere (unpublished observations). The original English version of the questionnaire was translated into Haitian Kreyòl and Spanish and then back-translated into English to compare to the original. After piloting, some basic changes were made to the questionnaire to ensure comprehension and comfort of participants. Household questionnaire responses were collected using Eagle Survey (v.1.3.3, The Carter Center, Atlanta, GA, USA) software on Samsung GalaxyTab3 tablets (Samsung, Seoul, South Korea).

Survey teams were composed of questionnaire administrators and laboratory technicians. Questionnaire administrators were Haitian-born and bilingual (Haitian Kreyòl, Spanish). Laboratory technicians were affiliated with the Dominican Ministry of Public Health (*Ministerio de Salud Pública*) and National Center for Control of Tropical Diseases (CENCET).

### Blood testing

Survey respondents and one other randomly selected individual of any age within the household were asked to provide a finger-prick blood sample for malaria and LF testing. Blood was collected in heparin-coated microtainer tubes and transported to local laboratory facilities for diagnostic testing the day of collection by CENCET scientists. Malaria was diagnosed by multi-species *Plasmodium* rapid diagnostic test (RDT; CareStart® HRP2/pLDH, Access Bio Inc., Somerset, NJ, USA) and blood smear microscopy, which is the gold standard diagnostic procedure in the DR. LF antigen was detected by filarial test strips (FTS; Alere, Inc., Scarborough, ME, USA). Follow-up confirmatory night blood testing for microscopic detection of microfilariae (mf) was conducted for all FTS-positive individuals. Any malaria- or LF-positive participant was offered treatment based on national guidelines.

### Data analysis

A total of 51 clusters in 44 unique *bateyes* were sampled across the three geographic strata. The raw dataset contained 1439 individuals from 780 distinct households. Electronic household questionnaire data were aggregated, exported to Excel format, and merged with laboratory results. Discrepancies between household and laboratory identification codes were reconciled by reviewing the original, paper data forms used by laboratory technicians. Twenty one laboratory samples and four household questionnaire observations could not be reconciled and were excluded from analysis. After data cleaning, a total of 1418 blood samples and 776 household questionnaires were included in the analysis.

Population-level estimates were calculated using Stata’s *svy* routine to account for the survey’s sampling weights, clustering effects, and stratification. To compare malaria knowledge between groups, a continuous outcome variable was created based on summation of correct responses to questions on transmission (range: 0−1), symptoms (0−6), and prevention behaviours (0−4); incorrect responses did not subtract from total score [27]. Significant differences (*P* < 0.05) in malaria knowledge scores were computed using *t*-tests. All analyses were done in Stata v.14.2 (version 14.2, College Station, TX, USA). The *subpop* command was used in analyses restricted to sub-groups.

## Results

### Malaria and LF prevalence

**Fig. 1:**
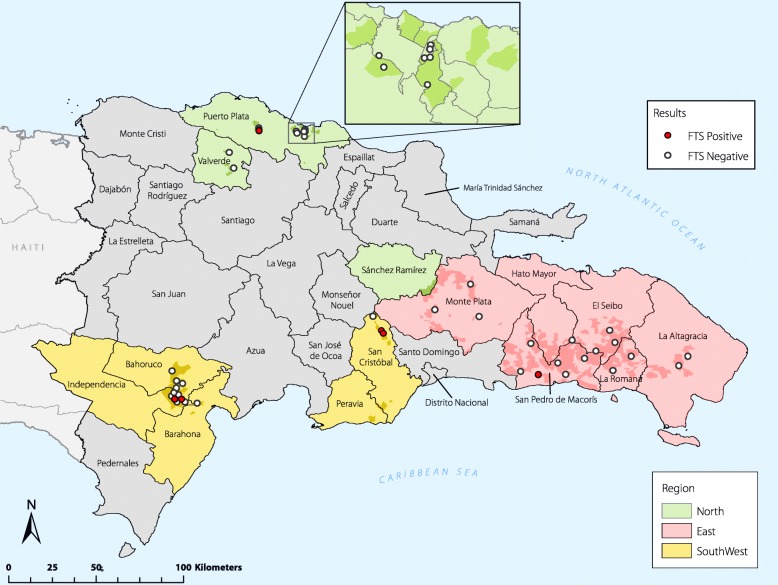
Map of the Dominican Republic showing the survey regions shaded in colour, with darker shading specifically illustrating *batey* locations, and filariasis test strip (FTS) results by sampled survey clusters (circles). Red circles indicate survey clusters with an FTS-positive individual; white circles indicate FTS-negative clusters

Of the 1418 individuals providing blood samples, 59% were female. Median age was 34 years (range 2−96). None (0%) of the samples were positive for *Plasmodium* infection by RDT or microscopy. Six individuals tested positive for LF antigen by FTS, resulting in a prevalence estimate of 0.5% (95% confidence interval [*CI*]: 0.2−1.5) across all three strata (Table 1). All FTS-positive individuals were microfilariae-negative in confirmatory night blood testing. Median age of FTS positives was 41.5 years (range, 24−66), with the two youngest (both age 24) born in Haiti. The six FTS-positive cases were geographically dispersed: one each in the North and East regions, and four in the Southwest—one each in Bahoruco and Independencia provinces and two in neighbouring clusters in San Cristobal province (Fig. 1). However, no single cluster contained more than one FTS-positive case. The case in the North was a migrant Haitian; the other five cases were permanent residents (having lived in the sampled *batey* for at least nine consecutive months in the previous year), with four of the five born in the DR. None of the FTS positive individuals reported travel to Haiti within the past 12 months.

**Table 1:** Prevalence of *Plasmodium* and *Wuchereria bancrofti* among *batey* residents, by survey region. Population estimates and 95% confidence intervals (*CI*) shown

	Southwest *n* = 453 % (95% *CI*)	North *n* = 481 % (95% *CI*)	East *n* = 484 % (95% *CI*)	Total *n* = 1418 % (95% *CI*)
*Plasmodium* spp. RDT and microscopy	0	0	0	0
*W. bancrofti* FTS	0.7 (0.2−2.1)	1.7 (0.5−5.8)	0.2 (0.0003−1.6)	0.5 (0.2−1.5)

*RDT* Rapid diagnostic test, *FTS* Filariasis test strip

### Household survey demographics

Table 2 displays demographic findings from the household questionnaire stratified by region with overall total *batey* estimates. The majority (84.1%) of respondents identified as the head-of-household. Mean respondent age across the three geographic strata was 44.4 years. Overall, a majority of respondents (54.1%) were female, with greater proportions of female than male respondents in the Southwest (66.0%) and North (70.3%) regions.

**Table 2:** Demographic characteristics, by survey region. Population estimates and 95% confidence intervals (*CI*) shown

Characteristic	Southwest *n* = 256 % (95% *CI*)	North *n* = 265 % (95% *CI*)	East *n* = 255 % (95% *CI*)	Total *n* = 776 % (95% *CI*)
Respondent category				
Head-of-household	84.2 (78.6−88.6)	88.3 (81.1−93.0)	83.2 (77.1−87.9)	84.1 (79.6−87.8)
Spouse	15.8 (11.4−21.5)	11.7 (7.0−18.9)	16.8 (12.1−22.9)	15.9 (12.2−20.4)
Age in years, mean (SE)	41.9 (1.1)	47.2 (4.2)	44.3 (1.1)	44.4 (1.2)
Sex				
Female	66.0 (57.4−73.5)	70.3 (65.4−74.7)	48.2 (42.4−54.1)	54.1 (48.8−59.4)
Male	33.8 (26.2−42.4)	29.7 (25.3−34.5)	51.8 (45.9−57.6)	45.9 (40.6−51.3)
Country of birth				
Haiti	18.5 (12.2−27.2)	23.6 (9.4−48.0)	50.9 (37.3−64.3)	42.5 (32.0−53.7)
DR	81.5 (72.8−87.9)	76.4 (52.0−90.6)	49.1 (35.7−62.7)	57.5 (46.3−68.0)
*If born in DR*:				
Haitian descent				
Yes	62.1 (43.2−77.9)	18.0 (7.6−36.8)	47.2 (36.1−58.6)	44.4 (34.0−55.3)
No	37.9 (22.1−56.8)	82.1 (63.2−92.4)	52.8 (41.4−63.9)	55.6 (44.7−66.0)
Language chosen for survey				
Spanish	48.2 (34.4−62.3)	66.8 (40.4−85.7)	35.1 (21.5−51.6)	41.8 (29.9−54.8)
Haitian Kreyòl	51.8 (37.7−65.6)	33.2 (14.3−59.6)	64.9 (48.4−78.5)	58.2 (45.2−70.1)
Documented	91.6 (85.4−95.2)	87.9 (78.2−93.6)	83.0 (76.9−87.8)	85.0 (80.2−88.7)
*By ethnicity:*				
Haitian-born	64.1 (47.7−77.7)	58.5 (34.4−79.1)	75.4 (64.4−83.8)	73.2 (64.4−80.5)
Dominican-born with Haitian descent	95.8 (90.7−98.2)	82.6 (58.7−94.0)	81.6 (64.7−91.4)	85.6 (74.2−92.5)
Dominican-born without Haitian descent	99.9 (98.7−100.0)	99.9 (98.7−100.0)	99.2 (93.7−99.9)	99.5 (96.9−99.9)
Residency				
Permanent resident (lived in *batey* for at least 9 consecutive months in last year)	93.2 (86.1−96.8)	88.3 (85.9−90.4)	82.8 (77.3−87.2)	85.1 (81.1−88.4)
Non-resident (migrant)	6.8 (3.2−13.9)	11.7 (9.6−14.1)	17.2 (12.8−22.7)	14.9 (11.6−19.0)
Travel to Haiti in last 12 months	8.7 (5.9−12.6)	13.0 (3.1−40.7)	8.7 (4.5−16.1)	9.4 (5.7−15.1)
Primary occupation				
Unemployed	39.3 (30.9−48.4)	46.3 (41.9−50.8)	33.7 (25.5−42.9)	36.4 (30.1−43.3)
Farmer	13.6 (8.7−20.8)	10.4 (4.9−20.4)	37.4 (27.8−48.3)	29.9 (22.2−39.0)
Homemaker/domestic work	24.5 (18.6−31.5)	8.0 (5.3−11.9)	8.3 (4.8−14.1)	10.5 (7.7−14.3)
Market vendor/retail/shop	11.2 (5.8−20.5)	20.5 (14.9−27.5)	10.3 (7.0−14.9)	12.0 (8.9−16.1)
Construction	2.6 (1.2−5.4)	8.6 (4.7−15.1)	2.4 (0.9−6.6)	3.4 (1.7−6.6)
Other	8.5 (5.5−12.8)	6.2 (2.1−17.0)	7.8 (3.5−16.5)	7.7 (4.2−13.5)

Abbreviation: *SE* Standard error, *CI* Confidence interval, *DR* Dominican Republic

Overall, a majority of respondents (57.5%) were born in the DR, which included those of Haitian- (25.5%) and non-Haitian descent (32.0%). Those born in Haiti therefore comprised the single largest ethnic group (42.5%). Some differences between survey regions were observed: *bateyes* of the East had the highest proportion of Haitian-born individuals (50.9%), while the North had the largest proportion of ethnic Dominicans (i.e. non-Haitian born or Haitian descended). In line with Haitian birth or descent, most participants (58.2%) completed the survey in Haitian Kreyòl rather than Spanish (41.8%).

Most respondents (85.0%) reported having some form of official documentation. When stratified by ethnicity, 73.2% of Haitian-born were documented compared to 85.8% of Dominican-born with Haitian descent and 99.5% of Dominican-born without Haitian descent.

Most respondents (85.1%) were considered permanent residents of their *bateyes* (having lived in the *batey* for at least 9 consecutive months in the previous year). Among ethnic groups, 23.2% of Haitian-born respondents were non-permanent (migrant) residents, compared to 14.1% of Dominican-born with Haitian descent and 4.8% of those without Haitian descent. Only a small percentage of respondents (9.4%) reported traveling to Haiti in the previous year.

Finally, respondents tended to be unemployed (36.4%), work in agriculture/farming (29.9%), do domestic work or live as homemakers (10.5%), or work as market vendors (12.0%). Differences in occupations were noted across the three regions. For example, 37.4% of respondents in the East described working in agriculture, compared to 10.4% in the North and 13.6% in the Southwest. Other occupations included moto taxi drivers, teachers, health or social service workers, and mechanics/technicians.

### Malaria intervention coverage

Nearly one-third (29.8%) of respondents overall said that they had been tested for malaria at home, though rates were higher in the Southwest (46.8%) and the North (49.8%) (Table 3). Approximately half (53.8%) of respondents reported owning a bed net, with ownership similar across survey regions. Most nets were obtained in another location within the DR, and ‘shop/market’ the most common source (81.3%). Overall, 82.3% of net owners said that they had slept under their nets the night before the survey. The most commonly cited reason for not sleeping under a net was that, ‘It was too hot’ (58.1%). Other reasons for not using a bed net in the previous night was that the net was too old, dirty, or torn (8.2%), it was not available (6.6%), no mosquitos or insects were in the area (5.9%), the net had a disagreeable odor (6.2%) and other (15%). Finally, 48.1% of residents stated that indoor residual spraying for mosquitos had occurred at their homes at some point in the previous 12 months. IRS coverage was notably different between the East (61.2%), Southwest (30.1%), and North (4.7%) regions.

**Table 3:** Malaria intervention coverage and practices, by survey region. Population estimates and 95% confidence intervals (*CI*) shown

Characteristic	Southwest *n* = 256 % (95% *CI*)	North *n* = 265 % (95% *CI*)	East *n* = 255 % (95% *CI*)	Total *n* = 776 % (95% *CI*)
Ever previously had malaria blood test at home				
Yes	46.8 (35.1−58.8)	49.8 (31.7−67.9)	22.1 (16.0−29.7)	29.8 (23.1−37.6)
No	50.1 (38.3−61.8)	49.4 (32.1−66.9)	76.3 (69.0−82.3)	68.5 (61.1−75.1)
Don’t know	3.2 (1.6−6.2)	0.8 (0.2−3.7)	1.6 (0.5−4.6)	1.7 (0.8−3.6)
Has mosquito net for self				
Yes	48.9 (32.3−65.7)	62.5 (52.6−71.5)	52.8 (43.1−62.3)	53.8 (46.0−61.3)
No	51.1 (34.3−67.7)	37.5 (28.5−47.4)	47.2 (37.7−56.9)	46.2 (38.7−54.0)
*If has mosquito net for self:*				
Area where obtained net				
In other community of DR	46.8 (30.3−64.0)	75.0 (55.0−88.1)	66.1 (54.7−75.9)	65.2 (55.6−73.7)
In this community	52.5 (35.3−69.1)	24.3 (11.7−43.6)	29.8 (22.3−38.6)	31.7 (24.7−40.0)
Haiti	0.8 (0.1−5.3)	0.6 (0.06−5.6)	4.1 (1.1−14.0)	3.1 (0.9−10.0)
Don’t know	0	0.1 (0.01−1.2)	0	0.02 (0.003−0.2)
*If has mosquito net for self:*				
Location where obtained net				
Shop/market	56.8 (43.5−69.3)	80.8 (76.5−84.5)	85.9 (77.5−91.5)	81.3 (75.0−86.3)
Mass distribution campaign	14.2 (8.7−22.4)	3.1 (0.9−10.7)	5.3 (2.0−13.6)	6.0 (3.1−11.4)
Family/friend	12.3 (6.6−21.7)	4.1 (0.9−16.3)	4.2 (1.7−10.2)	5.2 (2.8−9.2)
Healthcare setting	10.0 (2.7−29.9)	0.05 (0.005−0.5)	0	1.2 (0.30−5.1)
Other	6.9 (4.0−11.6)	12.0 (5.5−24.0)	4.0 (1.7−9.1)	5.8 (3.2−10.4)
Don’t know	0	0	0.70 (0.08−5.0)	0.05 (0.006−3.4)
*If owns mosquito net:*				
Slept under mosquito net last night				
Yes	80.3 (66.7−89.2)	86.6 (80.7−90.9)	81.5 (73.2−87.7)	82.3 (76.3−87.0)
No	19.8 (10.9−33.2)	13.4 (9.1−19.3)	18.5 (12.3−26.8)	17.7 (13.0−23.7)
*If did not sleep under net last night:*				
Reason:				
Too hot	26.5 (7.2−62.8)	75.5 (38.4−93.8)	61.1 (42.5−77.0)	58.1 (42.8−72.0)
Net too old, dirty, or torn	18.0 (6.7−40.1)	0	7.8 (1.7−28.9)	8.2 (2.8−21.7)
Net not available	0	0	9.1 (2.6−27.0)	6.6 (2.0−20.0)
Disagreeable smell of net	21.2 (5.3−56.6)	2.4 (0.3−19.6)	4.0 (0.6−22.4)	6.2 (2.1−16.8)
No mosquitos/insects here	25.8 (3.3−78.0)	1.7 (0.3−9.0)	2.8 (0.3−19.8)	5.9 (1.3−22.2)
Other	8.5 (1.8−32.1)	20.4 (4.5−58.0)	15.2 (6.9−30.5)	15.0 (7.8−26.7)
Indoor residual spray in home (within last 12 months)				
Yes	30.1 (20.1−42.6)	4.7 (1.4−14.4)	61.2 (45.1−75.1)	48.1 (35.4−61.0)
No	69.0 (56.8−79.0)	94.8 (84.0−98.4)	36.1 (22.2−52.8)	49.8 (36.5−63.1)
Don’t know	0.9 (0.02−3.6)	0.50 (0.10−2.8)	2.7 (1.3−5.6)	2.1 (1.1−4.3)

*CI* Confidence interval, *DR* Dominican Republic

### Malaria knowledge and related risk factors for vector-borne disease

Overall, less than half (44%) of the population had heard of malaria, with awareness among Haitian-born respondents (33.0%) notably lower than among Dominican-born without Haitian descent (57.8%). Of those who had heard of malaria, the most commonly reported symptom among all three groups was fever (57.8%), although 28.5% could not name a symptom. Most respondents (55.8%) cited mosquito bites as the primary cause of malaria, while 36.5% did not name a cause. Differences were not significant across ethnic groups in regards to reporting malaria symptoms or transmission source.

Sleeping under a mosquito net was the most commonly cited malaria prevention method (31.5%), followed by filling in puddles (9.6%) and avoiding mosquito bites (9.1%). Differences were not statistically significant across ethnic groups, though Haitian-born individuals and those of Haitian descent tended to cite sleeping under a net, filling in puddles/stagnant water and taking preventative medicine more than Dominicans without Haitian descent, whereas other methods tended to be more common among the latter group.

Working at night, which can lead to increased exposure to bites from malaria and LF-carrying mosquitos, was 16.0% overall and did not appear to differ significantly across ethnic groups, although working at night was less common among Dominican-born persons without Haitian descent.

Table 4 presents bivariate analysis of composite malaria knowledge scores. This score was based on correct answers to questions on malaria transmission, symptoms, and prevention (range, 0–11) and was applied to only those who had heard of malaria (*n* = 368). Mean scores were similar between male and female respondents. Those born in Haiti had the lowest mean score (2.4) followed by those born in the DR of Haitian descent (2.7) and Dominican-born without Haitian descent (2.9) (*P* < 0.001). Higher scores among those born in the DR—whether of Haitian descent or not (mean: 2.9)—were likely related to ability to read in Spanish, as scores were significantly higher among those who could read an entire sentence in Spanish (mean score: 3.0) vs. those who could not (mean score: 2.3) (*P* < 0.001). Malaria knowledge scores tended to be higher among permanent residents (2.8) versus non-permanent residents (2.1) (*P* = 0.06). Higher scores were also noted among those who do not work at night (2.8) versus those who do (2.0) (*P* = 0.014). Borderline differences were found between those who sought care for recent fever (2.9) versus those who did not seek care (1.8) (*P* = 0.066), while significant differences were not found between those who own mosquito net (2.7) versus those who do not (2.7) (*P* = 0.784) or between those who reported using the mosquito net last night (2.7) versus those who did not report using the net last night (2.7)  (*P* = 0.913).

**Table 4:** Bivariate analysis of malaria knowledge scores (range, 0–8) among respondents who had heard of malaria (*n* = 368)

Category	*n*	Mean (*SD*)	*t*	*P-value*
Sex				
Female	225	2.8 (0.12)	1.70	0.089
Male	143	2.5 (0.15)		
Ethnicity				
Haitian-born	92	2.4 (0.17)		
Dominican-born, Haitian descent	110	2.7 (0.29)	-5.46	< 0.001*
Dominican-born, no Haitian descent	166	2.9 (0.20)		
Read Spanish				
Can read entire sentence	209	3.0 (0.13)	-3.46	< 0.001*
Cannot read entire sentence	149	2.3 (0.14)		
Resident status in *batey*				
Permanent	340	2.8 (0.10)	-1.89	0.060
Non-permanent	28	2.1 (0.30)		
Works at night				
No	189	2.8 (0.13)	2.48	0.014*
Yes	35	2.0 (0.27)		
If had fever in last two weeks:				
Sought care for fever	30	2.9 (0.40)	-1.88	0.066
Did not seek care for fever	25	1.8 (0.42)		
If owns mosquito net				
No	148	2.7 (0.14)	-0.27	0.784
Yes	220	2.7 (0.13)		
*If owns mosquito net:*				
If used mosquito net last night				
No	39	2.7 (0.31)	-0.11	0.913
Yes	181	2.7 (0.14)		

* *P* value < 0.05

### Recent fever and treatment-seeking behaviour

Table 5 shows results of recent fever and treatment seeking behaviour stratified by ethnicity. Overall, 20% of respondents reported having a fever in the past two weeks (Table 5). Reported fever was higher among Haitian-born individuals (25.9%) and Dominican-born with Haitian descent (21.7%) compared to Dominican-born without Haitian-descent (11.5%). Among those who reported fever, a majority reported seeking care for it (56.0%), although care-seeking was lowest among those born in Haiti (46.2%).

**Table 5:** Recent fever and fever-related treatment-seeking of household survey respondents, by ethnicity. Population-level estimates and 95% confidence intervals (*CI*) shown

	Haitian-born	Dominican-born with Haitian descent	Dominican-born without Haitian descent	Total
	*n* = 256	*n* = 226	*n* = 290	*n* = 776
	%	%	%	%
	(95% *CI*)	(95% *CI*)	(95% *CI*)	(95% *CI*)
Reported fever in previous 2 weeks				
Yes	25.9 (19.1−34.1)	21.7 (13.2−33.6)	11.5 (5.7−21.7)	20.0 (15.3−25.8)
No	74.1 (65.9−80.9)	78.3 (66.4−86.8)	88.6 (78.3−94.3)	80.0 (74.1−84.6)
*If had fever*:				
Sought care for fever				
Yes	46.2 (25.6−68.1)	70.8 (51.2−84.9)	62.8 (35.7−83.7)	56.0 (40.3−70.5)
No	53.8 (31.9−74.4)	29.2 (15.1−48.8)	37.2 (16.3−64.3)	44.0 (29.5−59.7)
*If did not seek care for fever:*				
Reason for not seeking care				
Illness was not serious enough to seek care	53.2 (33.7−71.8)	71.1 (35.9−91.5)	88.4 (49.8−98.3)	61.8 (46.8−74.9)
Too expensive/cost	6.0 (1.5−21.4)	6.5 (1.4−25.6)	0	5.2 (1.7−15.1)
Hours of health centre are not convenient	0	3.5 (0.4−24.4)	9.0 (0.9−51.2)	2.0 (0.4−9.4)
I must work	1.8 (0.2−14.1)	0	0	1.2 (0.1−9.4)
Medical personnel do not treat me with respect	18.3 (7.3−38.7)	0	0	12.1 (4.5−28.9)
Health centre does not have staff	1.8 (0.2−14.1)	0	0	1.2 (0.1−9.4)
Lack of documents (any)	0.4 (0.04−3.4)	0	0	0.2 (0.03−2.2)
Other	2.8 (0.6−12.6)	11.9 (1.7−51.0)	2.6 (0.4−16.0)	4.4 (1.3−14.4)
Don’t know	15.7 (5.5−37.3)	7.1 (1.5−27.7)	0	11.8 (4.7−26.6)
*If sought care for fever*:				
Source of care				
Public hospital	72.5 (45.6−89.3)	76.1 (38.9−94.1)	70.9 (40.5−89.7)	73.4 (60.3−83.4)
Primary care (public) centre	18.5 (4.2−53.7)	5.9 (1.6−19.1)	7.5 (1.3−33.0)	11.9 (4.7−26.9)
Private hospital/clinic	5.6 (0.9−26.4)	7.7 (1.1−38.7)	0.5 (0.05−4.9)	5.3 (1.6−16.3)
Other	3.5 (0.8−13.3)	10.4 (2.3−35.8)	21.1 (4.9−58.4)	9.4 (3.4−23.4)
*If sought care for fever*:				
Received blood test for malaria				
Yes	24.9 (8.2−55.3)	41.1 (18.9−67.5)	24.7 (7.1−58.5)	30.5 (15.8−50.5)
No	69.4 (45.3−86.2)	58.9 (32.5−81.1)	75.3 (41.5−92.9)	67.0 (48.1−81.6)
Don’t know	5.7 (1.0−26.4)	0	0	2.5 (0.4−14.4)
*If had fever*:				
Took medicine for fever				
Yes	61.9 (44.7−76.5)	83.5 (66.1−93.0)	69.6 (40.4−88.5)	69.2 (54.5−80.9)
No	38.1 (23.5−55.3)	16.5 (7.0−33.9)	30.4 (11.5−59.6)	30.8 (19.1−45.5)

*CI* Confidence interval

The most commonly cited reason for not seeking care for the recent fever was that the illness was not considered serious (61.8%). To note, only Haitian-born (6.0%) and Haitian-descended (6.5%) respondents cited cost as a barrier to care; only Haitian-born respondents reported that, 'Medical personnel do not treat me with respect' (18.3%); and only Haitian-born individuals endorsed lack of documents as a reason for not seeking care.

The publicly-funded health system appeared to be the most preferred source for fever care. Of those who sought care for a recent fever, nearly three-quarters (73.4%) attended a public hospital, and another 11.9% went to a public primary care health centre. However, only 30.5% of those that sought care for a fever said that they received a blood test for malaria. A majority (69.2%) of those with a recent fever reported taking some type of medicine for the fever.

### LF Morbidity

Survey respondents also were asked to self-report LF morbidity. Overall, 7.1% (95% *CI*: 4.2–11.6%) reported having lymphedema within the past three months and 6.2% (95% *CI*: 2.4–14.8%) of men reported having an enlarged testicle. Lymphedema prevalence was similar across regions, while hydrocele was lowest in the North and highest in the East, but differences were not statistically significant.

## Discussion

This nationally representative survey of *bateyes* in the DR detected no evidence of active malaria or LF transmission, and a stable admixture of Haitian-born, Dominican-born of Haitian descent, and Dominican ethnicities with low levels of reported migration to and from Haiti.

Reported cases of confirmed malaria in the DR have declined 70% since 2010 to only 755 cases in 2016 [11]. Despite high-quality facility-based surveillance in the country, large cross-sectional malaria surveys have not been conducted recently. Previous risk maps implicated *batey*-dense regions, particularly the Southwest and East, as areas of relatively higher malaria transmission [4]. The estimated annual parasite incidence rates in 2015 for these two regions were 0.49 and 0.58 cases per 10,000 persons, respectively, compared to 0.01 case per 10,000 persons in the North [28]. However, this survey did not detect any RDT or microscopy-positive individuals, indicating minimal or even no malaria transmission within these rural areas. Indeed, urban transmission in Santo Domingo now accounts for the majority of cases nationally [28]. The decline in malaria in rural areas may be a result of active surveillance efforts in *bateyes* by the Dominican Ministry of Health. This is reflected in significant proportions of survey respondents in the Southwest and the East survey regions reporting a recent malaria blood test at home. The presence of vector control measures may also contribute to the decline in transmission, with half of survey respondents owning a bed net, and more than half of homes in the East sprayed with IRS in the past year. However, low (< 20%) bed net usage and low IRS coverage in the Southwest and the North highlight gaps in intervention coverage. Additional laboratory testing of filter paper specimens collected from survey participants is underway that includes polymerase chain reaction (PCR) analysis to detect low density *Plasmodium* infections, and serological analysis to determine evidence and timing of past exposure to malaria and LF among this population.

This survey also provides important information for LF elimination efforts in the DR by assessing prevalence across all age groups in both historically LF-‘endemic’ and ‘non-endemic’ areas. Two of the three LF-endemic foci in the country were included in this survey: the Southwest foci (which comprises *bateyes* in the three western-most provinces of Bahoruco, Barahona, and Independencia) and the East (*bateyes* in the five eastern-most provinces of El Seibo, Hato Mayor, La Altagracia, La Romana, and San Pedro de Macoris). Following the halt of MDA in the Southwest focus after 2007, post-treatment surveillance (PTS) surveys conducted in 2009 and 2012 revealed low (< 0.2%) antigen prevalence across all-age groups, with most positive individuals identified as non-permanent residents of the area [29]. In the present study, only two antigen-positive samples were detected in the three provinces of the Southwest LF focus (0.4% prevalence, 95% *CI*: 0.07−1.9%). The two individuals were older (age 46−66 years), amicrofilaremic, and permanent residents of near-by, but separate, survey clusters in different provinces, suggesting that these cases represent residual antigen persistence from past infections and that transmission likely remains interrupted in the Southwest focus. In the East LF focus, baseline prevalence was 1.4% in 2011 and one round of MDA (in 2014) had taken place by the time of this survey. Despite this, only one FTS positive individual was identified across all age groups, resulting in a prevalence estimate of 0.3% (95% *CI*: 0.03−2.2%) among the five-province LF East focus. Since then, two additional rounds of MDA have occurred, and a recent TAS-1 survey confirmed that prevalence among school-children 6−7 years old was significantly below the 2% prevalence threshold for MDA to stop (unpublished observations).

This survey also provides the first LF prevalence data in ‘non-endemic’ areas since baseline mapping was completed in 2007. A total of three FTS-positive individuals were identified in two ‘non-endemic’ provinces: Puerto Plata in the North region and San Cristobal in the Southwest survey region. Transmission in the North is unlikely as the individual was a non-permanent migrant born in Haiti, while one of the individuals in San Cristobal was also born in Haiti. It is likely that these individuals were exposed in Haiti, however the presence of a second antigen-positive sample in San Cristobal raises the possibility of an isolated transmission focus there. PELF plans to conduct confirmatory remapping surveys in 2019 across historically non-endemic areas, including San Cristobal, to determine whether there is evidence of transmission in areas not previously treated. Additionally, active surveillance follow-up in areas immediately surrounding FTS-positive individuals is recommended to assess the potential for residual transmission foci [30, 31]. This survey was not powered to exclude an upper 95% confidence limit of <2% in each individual region. However, the overall observed LF antigen prevalence was significantly lower than this hypothesized sustainable transmission level [32, 33]. Coupled with the failure to detect any microfilaria-positive samples, these results suggest that MDA has interrupted LF transmission in formerly endemic areas and active transmission is unlikely in other *bateyes* of the DR. Nonetheless, results also highlight the importance of continued post-treatment surveillance, particularly in areas with mobile populations, until transmission interruption is achieved across Hispaniola.

In addition to gaps in net coverage and indoor residual spraying (IRS), this survey identified other gaps in vector-borne disease interventions. Nearly 70% of the *batey* population reported never being tested for malaria at home. Furthermore, almost half of those with recent fever did not seek care, mainly because the fever was considered not serious enough. This implies that local, explanatory models of fever may downplay the importance of seeking care, which may be a hurdle for malaria elimination. Yet even among those with recent fever who did seek care (56%), less than one-third of them reported being tested for malaria at their care source, which was typically a public hospital or clinic. These findings suggest a need to improve diagnostic testing for malaria at the most commonly used points of care.

Historic associations of malaria and LF with areas of high concentration of Haitian migrant labourers have resulted in longstanding narratives of blame directed against Haitian and Haitian-descended people in the DR [34]. Cultural meanings of disease can change over time and reflect tensions about race, class, and morality [34–37]. In the DR, the malaria and LF elimination efforts transpire against a political backdrop that tends to denigrate the Haitian and Haitian-descended minority in the country [19, 21, 38]. Findings from this survey’s perceived discrimination module found that interpersonal experiences of discrimination were highest among Haitian-born respondents, followed by Dominican-born persons of Haitian descent (unpublished observations). Additionally, a qualitative study concurrent to this survey found that the 2013 *Sentencia* as well as poverty, discrimination, and limited social and economic mobility contributed to a diminished sense of social standing among *batey* residents [39]. High levels of perceived discrimination and feelings of not being recognized in society contrast with the fact that most respondents (85%) in this survey admitted to having some form of official documentation—meaning that despite having documents, people may still feel unrecognized or discriminated against. Such a high percentage of documented residents in *bateyes* may be due to the survey’s timing, which came nearly three years after the verdict. In the interim between the *Sentencia* and the survey, some undocumented residents may have moved elsewhere—certainly Haiti [40]—while others may have obtained new documents under the government’s regularization plan [21]. In a political and bureaucratic climate in which the validity of identity documents is always shifting, simply having documents does not necessarily impart secure standing in Dominican society. Of course, high self-reports of documentation may reflect response bias, in that some may have felt uncomfortable revealing their undocumented status.

In addition to higher levels of perceived discrimination, there appeared to be a consistent trend of increased risk for vector-borne disease among those with Haitian ancestry. Proportionally more Haitian-born and Dominican-born, Haitian-descended people reported recent fever compared to those without Haitian descent; proportionally more Haitian-born respondents did not seek care for their fever compared to the other two groups. While most agreed that not seeking care for recent fever was because, ‘The illness was not serious enough,’ it was nonetheless striking that nearly 20% of Haitian-born respondents said that they did not seek care because ‘Medical personnel do not treat me with respect,’ while no one from the other two groups cited that reason. Cost as a barrier to seeking care was cited only by Haitian-born and Haitian-descended people. Proportionally more Haitian-born respondents had never heard of malaria, did not know a symptom of malaria, did not know a prevention method, and had never received an at-home blood test for malaria compared to the other two groups. More Haitian-born individuals did not have a mosquito net for personal use compared to those born in the DR with and without Haitian descent, and more Haitian-born respondents did not sleep under their nets the night before the survey followed by those with Haitian descent and lastly those without Haitian descent. These differences in knowledge of vector-borne disease and preventive behaviours suggest different ways in which these groups understand malaria and varying degrees of exposure to public health campaigns in both Haiti and DR. Of course, these differences are also reflective of a larger pattern of health and socioeconomic disparities. Haitian migrants are likely undocumented, less educated, live and work in arduous conditions, and face a language barrier in the DR. Persons of Haitian descent born in the DR also face hurdles accessing educational and work opportunities, especially in the aftermath of the *Sentencia*. In essence, those most at-risk for vector-borne disease in the DR are also those who face greater degrees of structural violence in everyday life. Reaching this population, gaining their trust, and maintaining surveillance in their communities will be a challenge.

### Limitations

There are important limitations to this study, including the potential underestimation of malaria infection prevalence due to limited sensitivity of RDTs, which will be addressed through subsequent confirmatory polymerase chain reaction PCR testing. Secondly, despite the motivation to understand the risk of malaria among mobile populations within the DR, the survey surprisingly found a highly settled population currently living in *bateyes*. The survey was intentionally conducted during the *zafra* harvest period to provide the best opportunity of including migrant labourers. It is not known whether the household-based survey failed to capture this population or whether the role of migrant labourers simply has diminished with the decline of the sugar industry. Anecdotal reports indicate that migrants now mainly work in construction and other non-agricultural industries throughout the country. This suggests the present study results are credible and also highlight the need for additional surveys in other geographic areas to assess disease status among present day migrants. Third, despite the study team intentionally conducting survey activities during weekend evenings, when most household members were reported to be available, more females than males completed the household interview. It is possible that men were under-represented, perhaps because they were working at the time or had migrated elsewhere at the time of the survey. Finally, there is always potential for response bias among questionnaire participants due to nuances of language, power dynamics and rapport between respondents and interviewers, and other factors.

## Conclusions

Going forward, the final push towards elimination of both malaria and LF in the DR will focus increasingly on at-risk populations such as Haitian migrants, those living in *bateyes* and other rural areas, along with urban and peri-urban areas of Santo Domingo given the historic LF transmission and recent malaria outbreaks there [17, 28]. While malaria and LF now appear rare in *bateyes*, ongoing surveillance will be crucial to rapid detection of new cases, response to outbreaks, and prompt treatment to prevent transmission. Surveillance will in turn depend on strengthening relationships between community health workers, clinical care sites, and the general population. Some findings from this survey provide substance for reinforcing or developing new health promotion educative talks in the community. For example, given that the most common reason for not seeking care for recent fever was that, ‘The illness was not serious enough,’ health messages in higher-risk areas could underscore the importance of reporting fever to community health workers or otherwise seeking formal care, so that blood testing can detect possible infection.

The migrant population deserves special consideration for community engagement given their comparatively low level of knowledge about malaria and preventive behaviour, continued higher risk of importing malaria into the DR, and adverse social and political conditions that affect them. Furthermore, both Haitian migrants and Dominican-born persons of Haitian descent may harbour feelings of mistrust and disempowerment towards those in authority, given festering problems of discrimination and poverty. The national PELF represents one source of effective community engagement. Since 2002, this community-level program has worked extensively in *bateyes* across the country, collaborating with local authority structures, recruiting and training community health volunteers, and achieving high levels of participation in mass drug administration (MDA) campaigns [39, 41].

Finally, in the clinical setting, there is also room for improvement. While most of those with recent fever sought care (56%), most of them (67%) did not get tested for malaria at those care sites (typically within the public system), suggesting diagnostic and/or clinical limitations in care settings. Improving diagnostic capacity and clinical training, especially at sites that serve at-risk populations, will also be needed.

## Additional file

Additional file 1: Multilingual abstract in the five official working languanges of the United Nations. (PDF 484 kb)

## Abbreviations

*CI*: Confidence interval
DEC: Diethylcarbamazine citrate
DR: Dominican Republic
FTS: Filariasis test strip
IRS: Indoor residual spraying
LF: Lymphatic filariasis
MDA: Mass drug administration
PCR: Polymerase chain reaction
PELF: Program for elimination of lymphatic filariasis
PPS: Proportional to population size
PTS: Post-treatment surveillance
RDT: Rapid diagnostic test
