# Supplementary material for: Prevalence of malaria and lymphatic filariasis in bateyes of the Dominican Republic
Source: Infect Dis Poverty. 2019 May 27;8:39. doi: 10.1186/s40249-019-0547-3 (PMC6535845; doi:10.1186/s40249-019-0547-3)
Supplement: Supplementary file 1 — Multilingual abstract in the five official working languanges of the United Nations. (PDF 484 kb) [file 40249_2019_547_MOESM1_ESM.pdf]

Translation of the abstract into the five official working languages of the United Nations

انتشار الملاريا وداء الفيل الليمفاوي في مساكن الباتيّس في جمهورية الدومينيكان

هنتر م. كيز وغريغوري س. نولاند ومادسين بو دي روشار ومانويل غونزاليس

الملخص
خلفية: جزيرة هسبانيولا المشتركة بين هايتي وجمهورية الدومينيكان هي الجزيرة الوحيدة التي لا تزال مستوطنة بمرض الملاريا من جزر الكاريبي، وتتحمل وحدها 95% من عبء داء الفيل الليمفاوي في الأمريكتين. تسعى الدولتان إلى القضاء على هذين المرضين بحلول عام 2020. قد تُسبب الهجرة من هايتي، التي ينتشر بها كلا المرضين بنسبة أكبر، زيادة معدل انتقالهما في جمهورية الدومينيكان. تاريخيًا، يعيش العمال الهايتيون المهاجرون في "مساكن عمالية" زراعية تُدعى الباتيِّس، بريف جمهورية الدومينيكان. وقد خضع العديد من هذه المساكن لإعطاء جماعي للأدوية المضادة (Mass drug administration, MDA) للقضاء على داء الفيل الليمفاوي.
الطرق: من مارس إلى إبريل 2016، أجري استطلاع عرضي مقطعي لتحديد مدى انتشار الملاريا وداء الفيل الليمفاوي في أنحاء الباتيِّس بجمهورية الدومينيكان، بعد تقسيمها إلى ثلاث مناطق: الجنوب غربية والشمالية والشرقية. ومن خلال استبيان أسري (العدد = 776)، جُمعت البيانات الديموغرافية والأصول العرقية وأنماط التنقل وتغطية إجراءات التدخل المضادة للملاريا والمعرفة والحالات الحديثة للإصابة بحمى وحالات طلب العلاج. خضع فردان من كل أسرة (العدد = 1418) لاختبار الكشف عن طفيليات الملاريا بالفحص المجهري والاختبار التشخيصي السريع (Rapid Diagnostic Test, RDT) والكشف عن مستضد داء الفيل الليمفاوي بشريحة اختبار داء الفيل (Filariasis Test Strip, FTS).
النتائج: لم تكن نتائج أيٍّ من عينات الدم (0%) موجبة للبلازموديوم سواء بالفحص المجهري أو الاختبار التشخيصي السريع. كانت نتائج ستة أفراد موجبة لفحص شريحة اختبار داء الفيل (0.5%؛ فاصل الثقة (CI) 95%: 0.2-1.5)، ولكن لم يكن أيّ من هؤلاء (0%) موجبًا لوجود المكروفيلارية. ولد معظم سكان الباتيِّس في جمهورية الدومينيكان (57.8%)، وهم موثَّقون (85.0%) وذوو إقامة دائمة (85.1%). أقر عدد قليل جدًا من المدليين بالبيانات (9.4%) بالسفر إلى هايتي خلال العام الماضي. إجمالًا، كان نصف عدد المدليين بالبيانات (53.8%) يمتلك شبكة لتغطية السرير وقد أفاد 82.3% من ممتلكي الشبكات بأنهم استخدموها في الليلة السابقة. تفاوت استخدام رش المبيدات المترسبة الداخلي (Indoor Residual Spraying, IRS) حسب المنطقة (المدى: 4.7% - 61.2%). لجأ معظم الأفراد الذين أصيبوا بحمى حديثًا لطلب الرعاية الصحية (56.0%)، لكن لم يخضع لفحص الملاريا سوى 30.5% فقط ممن لجأوا للرعاية الصحية. ومقارنة بالفئات السكانية المولودة في جمهورية الدومينيكان، كثيرًا ما أبلغ المدليون بالبيانات هايتيو المولد عن إصابتهم بحالات حمى حديثة وبعدم لجوئهم للرعاية الصحية لعلاج الحمى وبعدم معرفتهم المسبقة بالملاريا وعدم قدرتهم على تسمية الأعراض أو طرق الوقاية.
الاستنتاجات: يبدو أن انتقال الملاريا وداء الفيل الليمفاوي منعدم أو شديد الانخفاض في مساكن الباتيِّس بجمهورية الدومينيكان، وهي مساكن تضم مزيج من السكان الهايتيين والدومينيكان. حالات السفر إلى هايتي نادرة، مما يعني أن خطر استيراد الملاريا وداء الفيل الليمفاوي منخفض. سيُمكن تحسين جودة رصد هذه الأمراض، تحديدًا بين الفئات المهمَّشة، وتعزيز القضاء عليها في كامل أنحاء الجزر من خلال مخاطبة الثغرات التي تم التعرف عليها في نطاق تغطية التدخلات المضادة للأمراض وفي المعرفة بالملاريا والسعي للحصول على العلاج وإمكانيات توصيل الخدمات.

Translated from English version into Arabic by Salma Anwar, Revised by Ghada Abaza, through

TRANSLATORS
WITHOUT BORDERS

## 多米尼加共和国 *bateyes* 地区中疟疾和淋巴丝虫病的患病率研究

Hunter M. Keys, Gregory S. Noland, Madsen Beau De Rochars and Manuel Gonzales

### 摘要

**引言：**伊斯帕尼奥拉岛是海地和多米尼加共和国共享岛屿。它是加勒比地区目前唯一的疟疾流行区，其淋巴丝虫病疾病负担占美洲的 95%。两国致力于 2020 年前消除这两种疾病。这两种疾病在海地的流行情况较为严重，来自这个国家的移民可能会加重上述两种疾病在伊斯帕尼奥拉岛上的传播。海地流动劳工常年居住在多米尼加农村中的“公司城镇”中，又称 *bateyes*。该地区已实施了大规模给药，以期消除淋巴丝虫病。

**方法：**2016 年 3 月至 4 月，为了确定多 *bateyes* 的疟疾和淋巴丝虫病的流行程度，我们在其西南，北和东部三个地区开展了一项横断面的聚类调查。家庭问卷（$n$ = 776）内容包括人口统计学、种族起源、流动模式、疟疾干预的覆盖度、知晓率以及发热和治疗情况。对每户的两个人进行检测（$n$ = 1418），其中用显微镜检查和快速诊断试剂检测疟原虫感染情况，用丝虫病试纸条来检测淋巴丝虫抗原。

**结果：**经显微镜或快速诊断检测，没有发现疟原虫阳性的血样（0%）。6 人为丝虫病试纸条阳性（0.5%; 95% *CI*：0.2 – 1.5），但没有人呈微丝蚴阳性（0%）。多数 *bateyes* 的居民都出生于多米尼加共和国（57.8%），记录在册人员占 85.0%，常住居民占 85.1%。在过去一年，少有受访者（9.4%）曾前往海地。总之，一半的受访者（53.8%）有蚊帐，其中的 82.3%在前一晚使用了蚊帐。除此之外，不同的地区室内残留喷洒情况不同，喷洒率从 4.7%到 61.2%不等。大多数近期发热的人员都接受了治疗（56.0%），但只有 30.5%的治疗患者接受了疟疾检测。与出生于多米尼加的人群相比，出生于海地的受访者近期发热情况更加频繁，更多的人未进行治疗、未听说过疟疾，也无法描述症状和预防方法。

**结论：**在多米尼加 bateyes 地区，居住着海地人和多米尼加人，疟疾和淋巴丝虫的传播水平较低。该地区的居民较少前往海地，因此疟疾和淋巴丝虫病输入的风险很低。解决该地区，尤其是边缘化人群中的干预范围、疟疾知晓、治疗和提供服务等方面的问题，将提高这些疾病的监测质量，并促进全岛上述疾病的消除进程。

Translated from English version into Chinese by Cong-Shan Liu, edited by Jin Chen

## Prévalence du paludisme et de la filariose lymphatique dans les *bateyes* de République dominicaine

Hunter M. Keys, Gregory S. Noland, Madsen Beau De Rochars et Manuel Gonzales

### Résumé

**Contexte :** Partagée entre Haïti et la République dominicaine, l’île d’Hispaniola est la dernière des Caraïbes où le paludisme est encore endémique, et concentre 95 % des cas de filariose lymphatique des Amériques. Haïti et la République dominicaine se sont fixé pour but d’éradiquer ces maladies

d'ici 2020. Les migrations depuis Haïti, où la prévalence des deux maladies est plus élevée, favorisent peut-être leur transmission en République dominicaine. Dans celle-ci, pour des raisons historiques, les travailleurs migrants haïtiens vivent dans des « villages ouvriers » agricoles appelés *bateyes*, dont beaucoup ont été visés par une administration de médicaments en masse (AMM) pour éliminer la filariose lymphatique.

**Méthodes :** Une étude transversale en grappes a été menée de mars à avril 2016 pour déterminer la prévalence du paludisme et de la filariose lymphatique dans les *bateyes* dominicains, stratifiés en trois régions: sud-ouest, nord et est. Un questionnaire par foyer (*n* = 776) a été utilisé pour relever les données démographiques, l'origine ethnique, les schémas de mobilité, la couverture par les interventions antipaludiques et la connaissance de celles-ci, ainsi que les épisodes récents de fièvre et les demandes de traitement. Deux personnes par foyer (*n* = 1418) ont été testées pour rechercher les parasites du paludisme par examen au microscope et test de diagnostic rapide (TDR) et l'antigène de la filaire par une bandelette réactive.

**Résultats :** Aucun échantillon de sang (0 %) n'a donné un résultat positif pour *Plasmodium* à l'examen microscopique ou au TDR. Les bandelettes réactives de six sujets étaient positives (0,5 % ; *IC* à 95 % de 0,2 à 1,5) mais aucun (0 %) des six n'était positif pour les microfilaires. La plupart des habitants des *bateyes* étaient nés en République dominicaine (57,8 %), résidents détenteurs d'un titre de séjour (85,0 %) et résidents permanents (85,1 %). Très peu des personnes interrogées (9,4 %) ont déclaré avoir voyagé en Haïti au cours de l'année écoulée. Dans l'ensemble, la moitié (53,8 %) des personnes interrogées possédaient une moustiquaire pour leur lit et 82,3 % de celles-ci ont répondu qu'elles l'avaient utilisé la nuit passée. Le taux de pulvérisations intérieures d'insecticide à effet rémanent était variable selon les régions (entre 4,7 % et 61,2 %). La plupart des personnes ayant récemment eu de la fièvre avaient demandé des soins (56,0 %), mais seulement 30,5 % des celles ayant demandé des soins ont fait l'objet d'un test de paludisme. Par rapport aux natifs de la République dominicaine, les sujets d'origine haïtienne ont été plus nombreux à rapporter un épisode de fièvre, à ne pas s'être fait soigner pour cette fièvre, à ne pas avoir entendu parler du paludisme et à ne pas pouvoir en citer les symptômes ni les mesures de prévention.

**Conclusions :** La transmission du paludisme et de la filariose lymphatique semble inexistante ou extrêmement faible dans les *bateyes* de République dominicaine, où cohabitent des habitants d'origine dominicaine et Haïtienne. Les voyages en Haïti sont rares, ce qui limite le risque d'importation des deux maladies. La correction des lacunes constatées dans la couverture par les interventions, la connaissance du paludisme et la prestation de services améliorera la qualité de la surveillance de ces maladies, en particulier parmi les populations marginalisées, et favorisera leur élimination dans toute l'île d'Hispaniola.

Translated from English version into French by Suzanne Assenat, Revised by Ahmad Dabaghzadeh, through

TRANSLATORS
WITHOUT BORDERS

**Распространенность малярии и лимфатического филяриоза в *батеях* Доминиканской Республики**

Хантер М. Киз (Hunter M. Keys), Грегори С. Ноланд (Gregory S. Noland), Мадсен Бо Де Рошар (Madsen Beau De Rochars) и Мануэль Гонсалес (Manuel Gonzales)

**Аннотация**

**Предпосылки:** остров Испаньола, принадлежащий Гаити и Доминиканской Республике (ДР), является единственным островом Карибского бассейна, где малярия остается эндемичным заболеванием и на долю которого приходится 95% всех случаев лимфатического филяриоза (ЛФ) в Южной и Северной Америке. Оба государства стремятся ликвидировать данные заболевания к 2020 году. Миграция с Гаити, где оба заболевания более распространены, может способствовать их передаче на территории ДР. Исторически рабочие-иммигранты из Гаити проживают в *батеях* - поселках при сахарных заводах в сельскохозяйственных регионах Доминиканской Республики, во многих из которых применялось массовое введение лекарственных препаратов для ликвидации ЛФ.

**Методы:** с марта по апрель 2016 г. проводилось перекрестное кластерное анкетирование населения *батеев*, которые были подразделены на три региона: юго-восток, север и восток. Целью анкетирования являлось определение распространенности малярии и ЛФ на территории *батеев*. Опросник для каждого домохозяйства ($n$ = 776) охватывал демографические данные, этническое происхождение, характер мобильности, степень охвата мероприятиями по борьбе с малярией, осведомленность о заболевании, недавнее наличие повышенной температуры и обращение за медицинской помощью. По два человека из каждого домохозяйства ($n$ = 1418) были обследованы на наличие малярийных паразитов с помощью быстрого диагностического теста (БДТ) и микроскопии и на наличие антигена ЛФ с помощью тест-полоски для выявления филяриоза.

**Результаты:** по результатам микроскопии и БДТ ни в одном (0%) из образцов крови не были обнаружены плазмодии малярии. Результат тест-полоски на выявление филяриоза был положительным у шести человек (0,5%; 95% *CI*: 0,2–1,5), однако ни у одного из них (0%) не было положительной пробы на наличие микрофилярий. Большинство жителей *батеев* были рождены в ДР (57,8%), официально зарегистрированы (85,0%) и имеют постоянный вид на жительство (85,1%). Малая часть респондентов (9,4%) сообщила о поездках в Гаити в течение последнего года. В общей сложности, половина респондентов (53,8%) имели москитные сетки для кровати, а 82% владельцев сеток использовала их накануне опроса. Применение опрыскивания помещений инсектицидами (ОПИ) варьировалось в зависимости от региона (диапазон: 4,7% - 61,2%). Большинство респондентов с недавней лихорадкой в анамнезе (56,0%) обращались за медицинской помощью, однако только у 30,5% из них были взяты анализы на малярию. По сравнению с уроженцами ДР большее количество респондентов, рожденных на Гаити, сообщало о недавнем повышении температуры, не обращалось за медицинской помощью в связи с этим, не слыхало о малярии и не могло назвать симптомы заболевания или способы его профилактики.

**Заключение:** Представляется, что передача малярии и ЛФ в доминиканских *батеях*, где проживает смешанное доминиканское и гаитянское население, отсутствует либо

присутствует в крайне низкой степени. Поездки на Гаити осуществляются редко, что означает низкую вероятность завоза малярии и ЛФ. Устранение выявленных пробелов в степени охвата населения мероприятиями по борьбе с малярией, осведомленность о заболевании и о необходимости обращения за медицинской помощью и оказание этой помощи улучшат контроль за данными заболеваниями, в частности, среди маргинализованных групп населения, и ускорят процесс ликвидации этих болезней на всей территории острова.

Translated from English version into Russian by Nataliya Zhydkikh, Revised by Alexander Somin through

TRANSLATORS
WITHOUT BORDERS

**La prevalencia del paludismo y la filariasis linfática en los *bateyes* de la República Dominicana**

Hunter M. Keys, Gregory S. Noland, Madsen Beau De Rochars y Manuel Gonzales

**Resumen**

**Antecedentes:** la isla La Española, territorio compartido entre Haití y la República Dominicana (RD), es la única isla restante en el Caribe en la que el paludismo es endémico y representa el 95 % de la carga de filariasis linfática (FL) en las Américas. Ambos países tratan de eliminar estas enfermedades antes de 2020. La migración procedente de Haití, donde ambas enfermedades son más prevalentes, podría fomentar la transmisión en la RD. Tradicionalmente, los trabajadores migrantes haitianos viven en pueblos rurales dominicanos llamados *bateyes* construidos por empresas agrícolas; muchos de ellos recibieron una administración masiva de medicamentos (AMM) para la eliminación de la FL.

**Métodos:** entre marzo y abril de 2016, se realizó una encuesta transversal por conglomerados para determinar la prevalencia del paludismo y la FL en los *bateyes* dominicanos estratificados en tres regiones: suroeste, norte y este. En un cuestionario del hogar ($n$ = 776) se recopilaron datos sobre demografía, origen étnico, pautas de movilidad, cobertura y conocimiento de la intervención contra el paludismo, así como fiebre y búsqueda de tratamiento recientes. Dos personas por hogar ($n$ = 1418) se sometieron a pruebas de detección de parásitos del paludismo por microscopía y pruebas de diagnóstico rápido (PDR) y del antígeno de FL mediante tira reactiva de filariasis (TRF).

**Resultados:** ninguna (0 %) muestra de sangre dio un resultado positivo para *plasmodium* ni por microscopía ni por PDR. Seis personas obtuvieron un resultado positivo en la TRF (0,5 %, *IC* del 95 %: 0,2-1,5), pero ninguna (0 %) de ellas fue positiva para microfilarias. La mayoría de los residentes de los *bateyes* nacieron en la RD (57,8 %), eran residentes documentados (85,0 %) y permanentes (85,1 %). Muy pocos encuestados (9,4 %) indicaron haber viajado a Haití en el último año. En general, la mitad (53,8 %) de los encuestados tenía un mosquitero y el 82,3 % de ellos señaló haberlo utilizado la noche anterior. La fumigación de interiores con efecto residual varió según la región (rango: del 4,7 % al 61,2 %). La mayoría de aquellos que sufrieron fiebre

recientemente buscó atención médica (56,0 %), pero solo el 30,5 % de ellos se sometió a pruebas de paludismo. En comparación con las poblaciones nacidas en la República Dominicana, los encuestados de origen haitiano indicaron con más frecuencia haber sufrido fiebre recientemente, no haber buscado atención para esta, no haber oído hablar del paludismo y no pudieron nombrar los síntomas ni los métodos de prevención.
**Conclusiones:** la transmisión del paludismo y de la FL parece estar ausente o ser extremadamente baja en los *bateyes* dominicanos, los cuales cuentan con una mezcla de residentes haitianos y dominicanos. Los viajes a Haití son poco frecuentes, lo que implica que el riesgo de importación del paludismo y de la FL es bajo. Abordar las deficiencias identificadas en la cobertura de la intervención, el conocimiento sobre el paludismo, la búsqueda de tratamiento y la prestación de servicios mejorará la calidad de la supervisión de estas enfermedades, especialmente entre las poblaciones marginadas, y fomentará la eliminación en toda la isla.

Translated from English version into Spanish by Mayra León, Revised by Lindsey Hoemann, through

TRANSLATORS
WITHOUT BORDERS
